# A case of multiple myeloma with plasma cell cannibalism and cytophagocytosis

**DOI:** 10.1002/jha2.194

**Published:** 2021-04-01

**Authors:** Julia Varghese, Habib Moshref Razavi

**Affiliations:** ^1^ Division of Haematology, Department of Medicine University of British Columbia Vancouver British Columbia Canada; ^2^ Division of Haematopathology and Transfusion Medicine Fraser Health Authority Vancouver British Columbia Canada; ^3^ Department of Pathology and Laboratory Medicine University of British Columbia Vancouver British Columbia Canada

**Keywords:** cannibalism, cytophagocytosis, multiple myeloma

A 60‐year‐old woman presented to our hospital complaining of fatigue, confusion, and left sided hip pain. At presentation, bicytopenia was noted with anemia and thrombocytopenia (77 g/L and 33 × 10^9^/L, respectively). She was also hypercalcemic with a new acute kidney injury (2.85 mmol/L and 370 micromol/L, respectively). Serum electrophoresis showed an IgG lambda monoclonal protein in the early gamma region (3.9 g/L) with background immunosuppression. The ratio of the lambda to kappa light chains was markedly elevated (8087 mg/L versus 182 mg/L). This was in conjunction with multiple new lytic lesions evident on her left iliac bone, right 8th rib, and L3 spinous process (first row, panel [A], abdominal computed tomography; a 4.7 cm destructive lesion in the iliac bone is marked). An urgent bone marrow biopsy was requested and performed. The aspirate (stained by May‐Grünwald‐ Giemsa x 50 objective panels B‐E) showed a large plasma cell infiltrate (85%). Plasma cells were moderately sized and showed abundance of blue/grey occasionally fraying cytoplasms. These cells also possessed round nuclear contours with relatively fine chromatins and occasional large nucleoli. Interestingly plasma cell cytophagocytosis was evident. Specifically cellular ingestion of platelets, orthochromic normoblasts (top row middle panel [B], black, and white arrows), erythrocytes (top row, third panel [C], white arrow; also note presence of Russel/Dutcher bodies black arrows, respectively), neutrophils (middle row, first panel [D]), and frank plasma cells (middle row and panel [E], grey arrow) were identified. A trephine biopsy showed a hypercellular bone marrow with a large plasma cell infiltrate. In situ cytophagocytosis was identified (Hematoxylin & Eosin stain, middle row third panel [F], white arrow). Cluster of differentiation (CD) 138, kappa, lambda stains showed a near subtotal replacement of bone marrow with lambda restricted plasma cells (bottom row panels (G‐I) respectively, x10 objective). Myeloma fluorescence in situ hybridization (FISH) showed the presence of *IGH/MAF* fusion (t(14;16)(q32.3;q23)), four copies of *FGFR3*, three copies of *CCND1*, and relative loss of *TP53*. Cytogenetic studies showed a complex karyotype: 79–81,XX,add(1)(q21)x2,+2,+2,+3,+3,+5,+5,der(6)t(6;7)(q21;q11.2),+7,+9,+10,+10, del(11)(q21q23),+add(11)(p15)x2,del(12)(q13)x2,add(14)(q32)x2,+add(15)(p11.2)x2,+add(16)(p11.1),+17,+del(17)(p11.2),+18,+18,+19,+19,+20,+20,+21,+21,+der(22)t(4;22)(q12;p11.2)x2,+der(?)t(1;?)(q21;?)x2,+mar1,+mar2,+mar3,+mar4[cp7]/83,XX,add(1)(q21)x2,+3,+3,+5,+5,add(6)(q25)x2,+7,+7,add(8)(q13)x2,add(8)(q22)x1‐2,+9,+9,+10,+10,+add(11)(p15)x2,+add(12)(q15)x2,add(14)(q32)x2,+15,+15,+add(16)(p11.1)x2,+17,+del(17)(p11.2),+18,+18,+19,+19,+19+20,+20,+21,+21,+der(22)t(4;22)(q12;p11.2)x2,+der(?)t(1;?)(q21;?)x2,+mar1,+mar2,+mar3,+mar4[cp3]nuc ish(TP53 × 2,D17Z1 × 3)[42/100]/(TP53 × 2,D17Z1 × 4)(52/100),(FGFR3 × 4,IGHx2)[77/100],(CCND1 × 3,IGHx2)[85/100],(IGHx2,MAFx4)(IGH con MAFx2)[77/100].

As her initial blood film showed mild schistocytosis, and with renal injury and confusion, diagnosis of thrombotic thrombocytopenic purpura was considered. However, her haptoglobin and ADAMTS‐13 levels were normal, and her red cell fragmentation was attributed to hemophagocytosis. Following the diagnosis of R‐ISS stage III lambda light chain multiple myeloma, the patient was treated with three cycles of cyclophosphamide, bortezomib, and dexamethasone therapy. As well, a melphalan‐conditioned autologous stem cell transplant is scheduled.

Extremely rare, myeloma‐related hemophagocytosis has not been associated with a specific immunoglobulin or light chain subtype. Although the presence of identifiable cytogenetic abnormalities is consistent with a more proliferative potential, it is unclear whether an association with phagocytosis exists. Speculation about the utility of aberrant expression of CD15 and CD56 in plasma cells phagocytosis is present in the literature. While the exact mechanism of phagocytosis is not clear, expansion of B cell clones with innate phagocytic
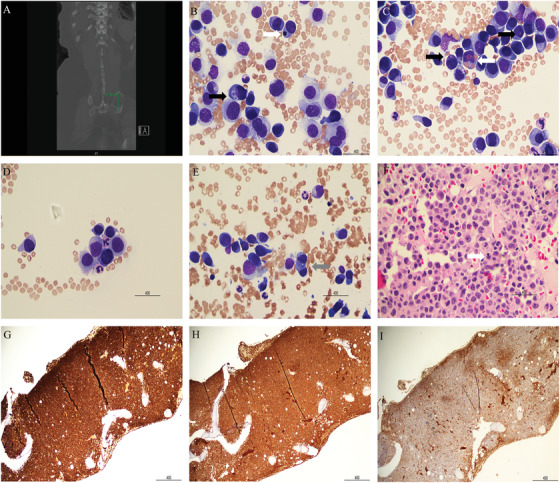
 potential has been considered. Notwithstanding this proposition needs to be confirmed.

